# The immunosuppressive role of CCR8^+^ Tregs in gastric cancer

**DOI:** 10.7150/jca.121363

**Published:** 2025-11-03

**Authors:** Koichi Jinushi, Yoshinori Hayashi, Koichi Morishita, Takuro Saito, Atsunari Kawashima, Yuichiro Doki, Azumi Ueyama, Hisashi Wada

**Affiliations:** 1 Department of Clinical Research in Tumor Immunology, Graduate School of Medicine, Osaka University, Suita, Osaka 565-0871, Japan.; 2 Department of Gastroenterological Surgery, Graduate School of Medicine, Osaka University, Suita, Osaka 565-0871, Japan.; 3 Pharmaceutical Research Division, Shionogi & Co., Ltd., Toyonaka, Osaka 561-0825, Japan.; 4 Department of Urology, Graduate School of Medicine, Osaka University, Suita, Osaka 565-0871, Japan.

**Keywords:** gastric cancer, CCR8⁺ regulatory T cells, tumor microenvironment, spatial analysis, anti-CCR8 therapy

## Abstract

**Background:** Gastric cancer remains one of the most common causes of cancer death, with a notably high incidence in East Asian countries. Regulatory T cells (Tregs) suppress antitumor immunity in the tumor microenvironment, and recent studies have identified C-C motif chemokine receptor 8 (CCR8) as a selective marker for tumor-infiltrating activated Tregs. However, the role of CCR8⁺ Tregs in gastric cancer remains unclear.

**Methods:** This study retrospectively analyzed 80 gastric cancer patients who underwent curative resection. Immunohistochemistry dual staining for CCR8/Foxp3 and granzyme B/CD8 was performed, followed by automated image analysis and spatial profiling. The correlation between CCR8⁺ Tregs and CD8⁺ T cells, as well as their prognostic significance, was evaluated.

**Results:** CCR8⁺ Treg density positively correlated with CD8⁺ T cell infiltration. However, a low CD8⁺ T cell/CCR8⁺ Treg ratio was significantly associated with worse recurrence-free survival (P = 0.023). Reduced granzyme B expression was observed in CCR8⁺ Treg-dense hotspots, suggesting the presence of a localized immunosuppressive environment. Spatial analysis revealed that CCR8⁺ Tregs were preferentially localized at the tumor invasion front. Furthermore, distance analysis showed that fewer CD8⁺ T cells were present around CCR8⁺ Tregs than around CCR8⁻ Tregs, suggesting a localized immunosuppressive effect that may restrict CD8⁺ T cell proliferation.

**Conclusions:** Our findings suggest that CCR8⁺ Tregs suppress antitumor immunity in gastric cancer by affecting surrounding CD8⁺ T cells through spatial segregation. Targeting CCR8⁺ Tregs may offer a promising strategy to improve the efficacy of immunotherapy in gastric cancer.

## Introduction

Among all cancers worldwide, gastric cancer is the fifth most frequently diagnosed and the fifth leading cause of cancer death 1. The incidence and mortality rates of gastric cancer are high in East Asia, with Japan having the highest incidence rate 2,3. In cases of unresectable advanced or recurrent gastric cancer, chemotherapy is the primary treatment. Recent advancements have led to significant tumor reduction effects. However, a complete response to chemotherapy remains rare, and clinical trial results from domestic and international studies indicate that the median overall survival is approximately 10 to 20 months 4-6. The introduction of immune checkpoint inhibitors (ICIs), such as anti-PD-1/PD-L1 and anti-CTLA4 antibodies, has significantly improved the prognosis of gastric cancer patients 7,8. However, the response rate to ICI monotherapy is as low as 11%, and its therapeutic efficacy remains limited 8. Therefore, elucidating the tumor microenvironment of gastric cancer and identifying molecular targets that can enhance the efficacy of ICIs may lead to the development of novel therapeutic strategies.

To escape immune detection, tumor cells employ various strategies, such as recruiting immunosuppressive cells, releasing inhibitory cytokines, and expressing immune checkpoint ligands 9. Among these, regulatory T cells (Tregs) have garnered particular attention as a major immunosuppressive cell subset that inhibits antitumor immunity. Tregs are a subset of CD4^+^ T cells that express the transcription factor Foxp3, which regulates various immune cells to maintain immune homeostasis and tolerance 10. In the tumor microenvironment (TME), activated Tregs with strong suppressive activity accumulate and impair the function of cytotoxic T cells, allowing cancer cells to escape immune attack 11. Therefore, modulating Tregs may activate antitumor immunity, suggesting Tregs might be a promising target to enhance antitumor responses. C-C motif chemokine receptor 8 (CCR8) has emerged as a molecule of particular interest in this field. Identified via transcriptomic profiling and flow cytometric analysis, CCR8 was shown to be selectively expressed on regulatory T cells that infiltrated tumors 12-14. CCR8^+^ Tregs are considered a highly immunosuppressive subset of activated regulatory immune cells with strong antitumor immune suppression activity 15. Preclinical studies using mouse tumor models have demonstrated that the depletion of CCR8^+^ Tregs through anti-CCR8 antibody administration resulted in significant tumor shrinkage 14,16-21. The antibody exerts its effect by enhancing CD8⁺ T cell activity, as evidenced by elevated levels of granzyme B (GzmB) and interferon-γ following its administration 14,17. Our recent analyses of lung cancer patients suggested that the accumulation of CCR8^+^ Tregs was associated with the impaired cytotoxic function of neighboring CD8^+^ T cells and a poor prognosis 16,22.

This study investigated whether CCR8^+^ Tregs contribute to the formation of an immunosuppressive environment in gastric cancer by evaluating the correlation between tumor-infiltrating CCR8^+^ Tregs and CD8^+^ T cells in human gastric cancer patients, as well as assessing their clinical background and prognosis.

## Materials and Methods

### Retrospective identification of patients and data collection

Patients diagnosed with gastric cancer who underwent curative resection at Osaka University Hospital between January and December 2016 were included in the study. Cases with distant metastasis, synchronous or metachronous malignancies, remnant gastric cancer, recurrent gastric cancer, or those who had undergone endoscopic submucosal dissection before surgery were excluded. Data from 80 patients were analyzed retrospectively, including clinical and pathological features, surgical details, and clinical outcomes, based on hospital records and pathology reports. Tumor staging followed the union for international cancer control TNM classification (8th edition) 23. All patients were monitored for recurrence and survival, and those without recurrence were followed up for up to five years postoperatively. The study protocol was reviewed and approved by the Ethics Committee of Osaka University Hospital (approval number. 13266) and adhered to the ethical guidelines set forth in the Declaration of Helsinki. Given the retrospective nature of the study, informed consent was obtained through an opt-out process.

### Double staining for immunohistochemistry (IHC) analysis

For each case, surgical tissue samples were fixed in formalin and embedded in paraffin. Three consecutive sections (4-µm thick) were obtained and stained with hematoxylin and eosin, and double immunostaining targeting CCR8/Foxp3 and GzmB/CD8 was performed. Immunostaining was conducted using the BOND RX automated IHC system (Leica Microsystems, Wetzlar, Germany), following the procedures outlined for the BOND Polymer Refine and HRP Plex Detection Kits. First, tissues were deparaffinized using BOND Dewax Solution and pretreated at 100°C for 20 minutes with Epitope Retrieval Solution 2 (EDTA buffer, pH 9.0). Diluted anti-CCR8 or -CD8 antibody was applied to the sections and incubated at room temperature for 15 minutes. After incubation with rabbit anti-mouse IgG, Polymer at room temperature for 8 minutes, anti-rabbit poly-HRP-IgG was applied and incubated for another 8 minutes. Peroxidase blocking was performed using a Peroxidase Block reagent at room temperature for 5 minutes. The tissues were then developed by reacting them with 3,3′-diaminobenzidine tetrahydrochloride hydrate at room temperature for 10 minutes. Subsequently, antigen retrieval was performed again using Epitope Retrieval Solution 1 at 100 °C for 20 minutes. Then, anti-Foxp3 or anti-GzmB antibody was applied and incubated at room temperature for 15 minutes. The Post Primary reagent was added and incubated at room temperature for 8 minutes, followed by addition of the Polymer reagent for another 8 minutes. The tissues were then developed using a Blue chromogen for Foxp3 staining and a Green chromogen for GzmB staining at room temperature for 8 minutes. The sections were counterstained with hematoxylin, air-dried at 37 °C for 30 minutes, and mounted on glass slides. The primary antibodies used were as follows: CCR8 (433H, mouse monoclonal, BD Biosciences, Franklin Lakes, NJ, USA, 0.15 μg/mL), CD8 (4B11, mouse monoclonal, Leica Microsystems GmbH, Wetzlar, Germany, 1:3 dilution), Foxp3 (236A/E, mouse monoclonal, Abcam, Cambridge, UK, 5 μg/mL), GzmB (11F1, mouse monoclonal, Leica Microsystems GmbH, Wetzlar, Germany, 1:3 dilution).

### Analysis of IHC images

Tissue sections subjected to double staining were scanned at 20× magnification using the NanoZoomer S60 slide scanner (Hamamatsu Photonics, Shizuoka, Japan), which produced digital slide images. These images were analyzed with HALO software (version 3.5, Indica Labs, Albuquerque, NM, USA), which enabled automated processes including annotation, algorithm training, and quantitative cell analysis. Cell quantification was performed using an algorithm trained during the analysis workflow. Tumor regions were delineated by an experienced pathologist based on corresponding hematoxylin and eosin-stained slides. Positive cell counts within tumor areas were evaluated using either the Whole Tumor Area (WTA) or Region of Interest (ROI) protocol, as detailed below:

[Treg (cells/mm²)] = Foxp3^+^ cell counts per unit area

[CCR8^+^ Treg (cells/mm²)] = CCR8^+^ Foxp3^+^ cell counts per unit area

[CD8^+^ T cell (cells/mm²)] = CD8^+^ cell counts per unit area

[GzmB^+^ CD8^+^ T cell (cells/mm²)] = GzmB^+^ CD8^+^ cell counts per unit area

[%CCR8^+^ in Treg] = Percentage of CCR8^+^ Foxp3^+^ cells among total Foxp3^+^ cells

[%GzmB^+^ in CD8^+^ T cell] = Percentage of GzmB^+^ CD8^+^ cells among total CD8^+^ cells

For the WTA protocol, the analysis was conducted across the entire tumor region within each section. In contrast, the ROI protocol involved generating cell density heatmaps from CCR8/Foxp3-stained images using HALO's spatial analysis function. Fields measuring 500 × 500 μm with the highest positive cell counts were identified, and the five most enriched areas were selected as “hot spots” for further analysis. These selected regions were then matched and analyzed across both CCR8/Foxp3- and CD8/GzmB-stained slides.

For cell distance analysis, CCR8^+^ Tregs and CCR8^-^ Tregs were used as starting points, and the number of the nearest CD8^+^ T cells or GzmB^+^ CD8^+^ T cells was evaluated in increments of 10 μm using the WTA protocol. In the analysis of clinical characteristics and prognosis, measurements obtained from the whole-tissue analysis using the WTA protocol were used.

### Statistical analysis

All statistical analyses and descriptive evaluations were carried out using JMP Pro version 16.2.0 (SAS Institute, Cary, NC, USA) and GraphPad Prism version 10.3.1 (GraphPad Software, Boston, MA, USA). Correlations were assessed via linear regression analysis. For group comparisons, the Mann-Whitney *U*-test and Fisher's exact test were used for continuous and categorical variables, respectively. Recurrence-free survival (RFS) was analyzed using Kaplan-Meier curves, with significance determined by the log-rank test. Hazard ratios (HRs) with 95% confidence intervals (CIs) were calculated using the Cox proportional hazards model. A p-value less than 0.05 was considered statistically significant.

## Results

### Counts of infiltrating CCR8^+^ Tregs and CD8^+^ T cells in the WTA

**Figure 1:**
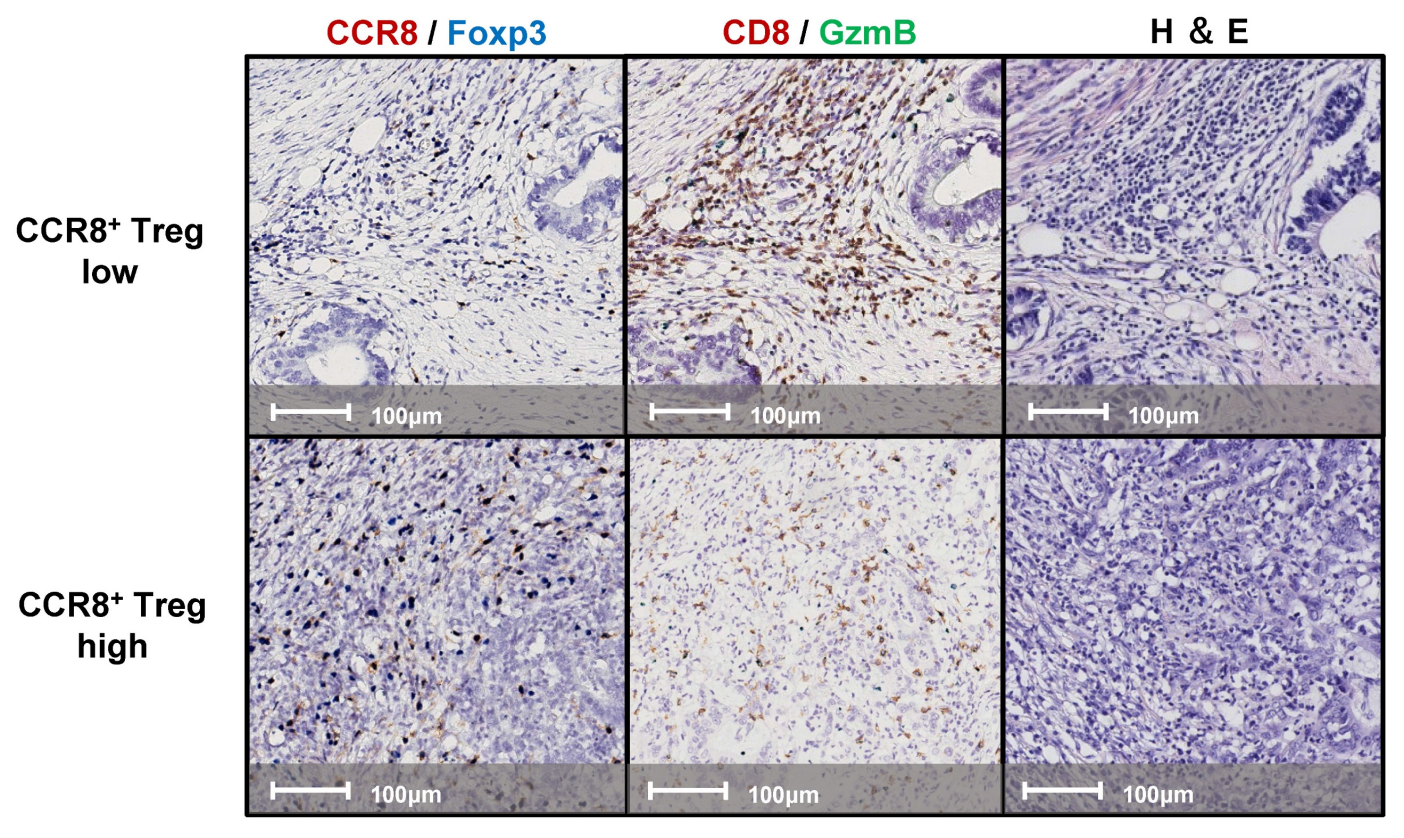
Representative images of double-stained immunohistochemistry assays. Lesions with high (upper panels) and low (lower panels) CCR8^+^ Treg infiltration were stained with CCR8 (brown) and Foxp3 (blue); left panels, CD8 (brown) and GzmB (Green); middle panels, and hematoxylin and eosin (H&E); right panels.

**Figure 2:**
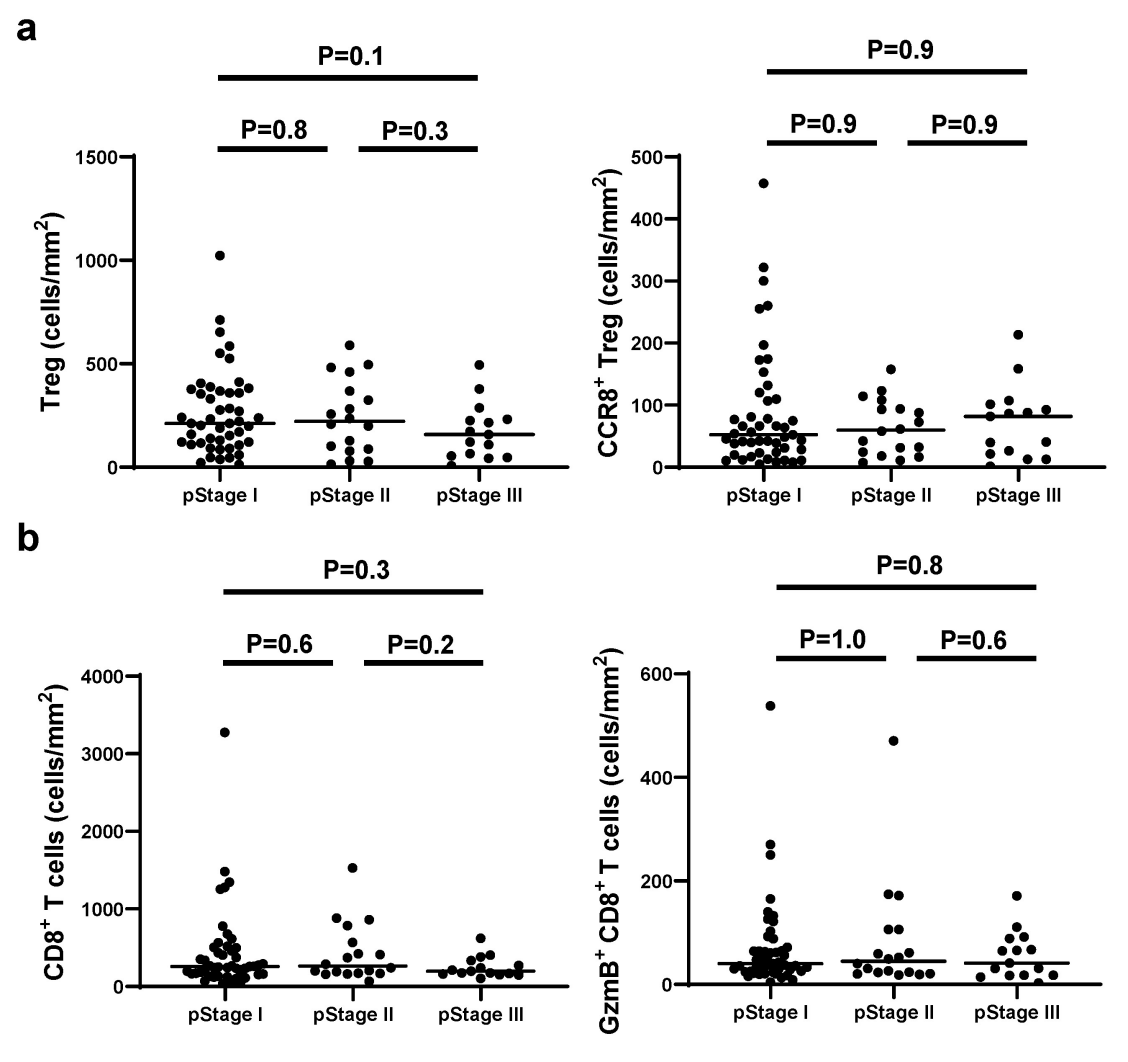
Comparison of CCR8^+^ Treg and GzmB^+^ CD8^+^ T cell density by pathological stage. **a.** The numbers of total Tregs and CCR8^+^ Tregs per area by pathological stage are shown. **b.** The numbers of total CD8^+^ T cells and GzmB^+^ CD8^+^ T cells per area by pathological stage are shown.

We examined continuous tissue sections from 80 gastric cancer patients using dual staining for CCR8/Foxp3 and GzmB/CD8. Figure 1 presents representative IHC images of two typical cases with high and low levels of CCR8^+^ Treg infiltration. First, we used the WTA protocol to evaluate the infiltration of Tregs, CCR8^+^ Tregs, CD8^+^ T cells, and GzmB^+^ CD8^+^ T cells to the whole tumor area. The results showed no statistically significant differences in the numbers of [Tregs (cells/mm²)], [CCR8^+^ Tregs (cells/mm²)], [CD8^+^ T cells (cells/mm²)], and [GzmB^+^ CD8^+^ T cells (cells/mm²)] across different pathological stages (pStages) (Figure 2), whereas the [%CCR8^+^ in Tregs] was significantly higher in Stage III compared with Stage I (*P* = 0.03) (Figure S1). Next, we investigated the relationship between CCR8^+^ Treg infiltration, CD8^+^ T cell infiltration, and GzmB expression in CD8^+^ T cells. The results showed that [CCR8^+^ Treg (cells/mm²)] positively correlated with [Treg (cells/mm²)] and [CD8^+^ T cells (cells/mm²)], suggesting that tumors with a high number of CCR8^+^ Tregs also have more CD8^+^ T cells. However, a weaker positive correlation was observed between [CCR8^+^ Tregs (cells/mm²)] and [GzmB^+^ CD8^+^ T cells (cells/mm²)]. Conversely, tumors with a high number of CCR8^+^ Tregs tended to have lower [%GzmB^+^ in CD8^+^ T cells] (Figure S2).

### Survival analysis according to CCR8^+^ Tregs and CD8^+^ T cell infiltration

**Figure 3:**
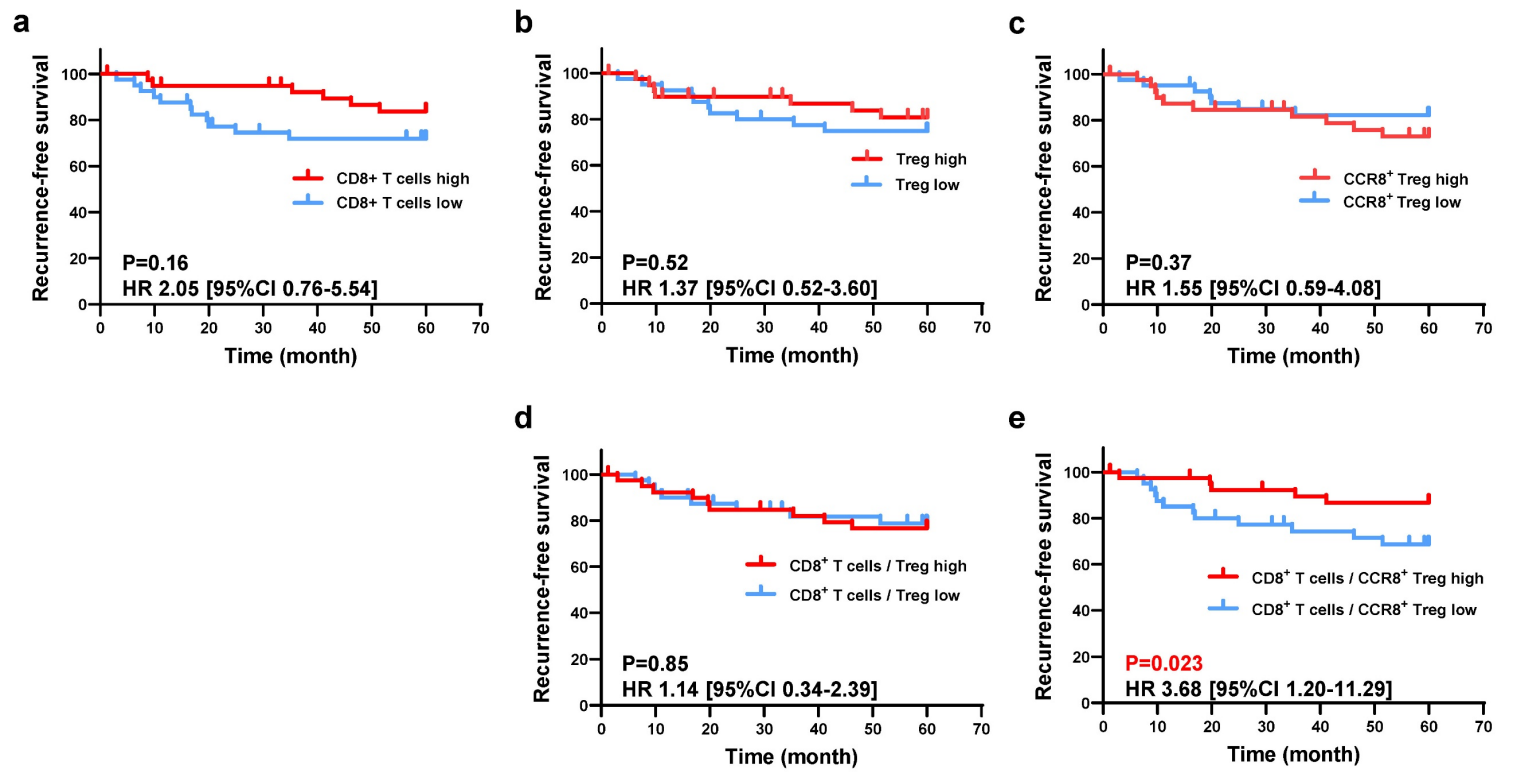
Evaluation of the prognostic impact of CCR8^+^ Treg and CD8^+^ T cell infiltration. **a.** Patients were divided into high-density and low-density groups based on the median CD8^+^ T cell density, and the 5-year survival rates were compared between the two groups. The comparison was also performed based on the density of Tregs** (b)** and the density of CCR8^+^ Tregs** (c)**. **d.** To evaluate the mutual prognostic impact of Tregs and CD8^+^ T cells, patients were similarly divided into two groups based on the ratio of CD8^+^ T cell density to Treg density, and the 5-year survival rates were compared between the two groups. **e.** Patients were similarly divided into two groups based on the ratio of CD8^+^ T cell density to CCR8^+^ Treg density, and the 5-year survival rates were compared between the two groups.

Next, we investigated the relationship between the immune profile observed in the histological analysis and RFS. Using the WTA protocol, all patients were divided into two groups based on the median values of [Treg (cells/mm²)], [CCR8⁺ Treg (cells/mm²)], [CD8⁺ T cells (cells/mm²)], and [GzmB⁺ CD8⁺ T cells (cells/mm²)], and their respective RFS was compared. As shown in Figure 3, the low [CD8⁺ T cells (cells/mm²)] group had worse RFS than the high group (5-year RFS: 71.8% vs. 83.7%, *P* = 0.16), and the high [CCR8⁺ Treg (cells/mm²)] group had worse RFS than the low group (5-year RFS: 72.9% vs. 82.1%, *P* = 0.37), but neither result was statistically significant. However, the low CD8⁺ T cell/CCR8⁺ Treg ratio group had significantly worse RFS compared with the high ratio group (5-year RFS: 66.3% vs. 89.2%, P = 0.023), whereas there was no significant difference in RFS between the low and high CD8⁺ T cell/Treg ratio groups (5-year RFS: 76.7% vs. 78.8%, P = 0.85) (Figure 3). ​When comparing clinicopathological characteristics, the group with a low CD8⁺ T/CCR8⁺ Treg ratio exhibited a significantly lower differentiation rate (47.5% vs. 72.5%) and a significantly higher rate of venous invasion (47.5% vs. 22.5%) compared to the high CD8⁺ T/CCR8⁺ Treg ratio group (Table 1). Additionally, when patients were classified into two groups based on [%CCR8⁺ in Tregs], the high [%CCR8⁺ in Tregs] group had significantly worse RFS than the low group (5-year RFS: 62.7% vs. 92.2%, *P* = 0.0022). When patients were stratified further by pStage and classified into two groups based on [%CCR8⁺ in Tregs], although no statistically significant difference was observed, RFS was worse in the high [%CCR8⁺ in Tregs] group compared with the low group across all stages (Figure S4). These results indicate that the impact of CCR8⁺ Tregs on CD8⁺ T cells was related to poor prognosis of patients with gastric cancer.

### Correlation between CCR8^+^ Tregs and GzmB^+^ CD8^+^ T cells in the ROI

**Figure 4:**
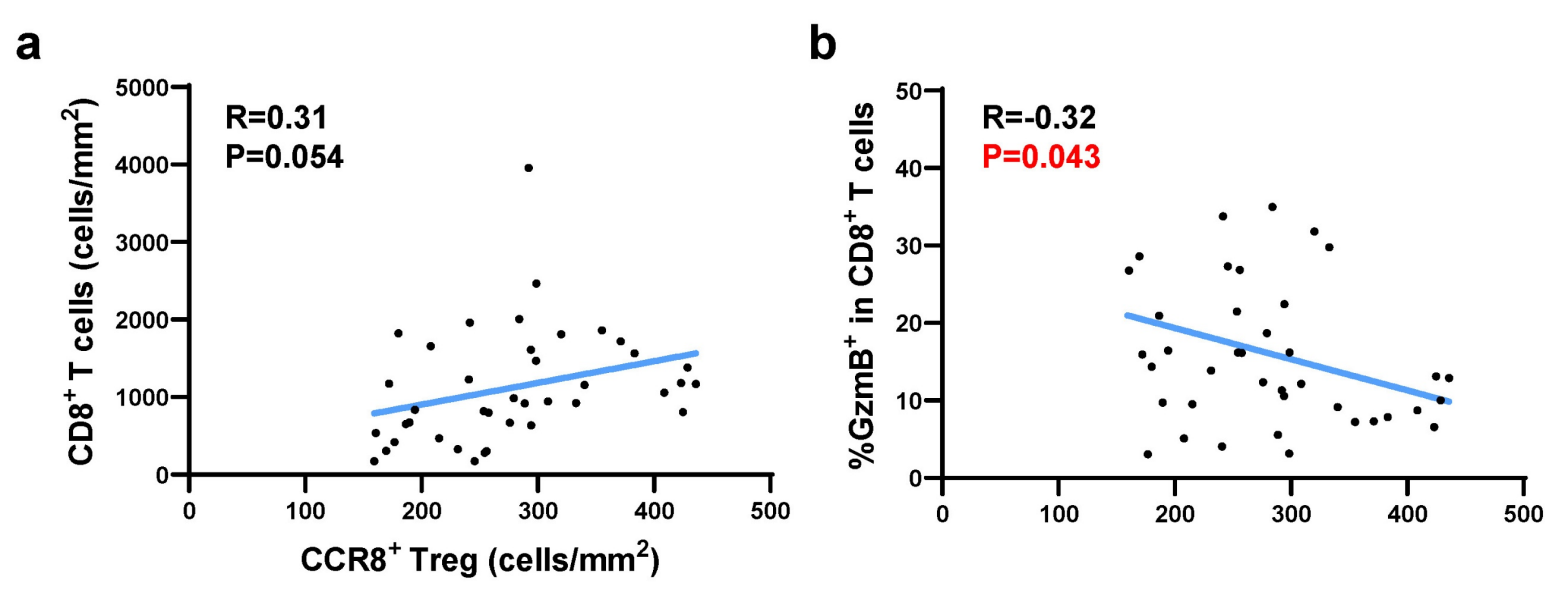
The correlation between CCR8^+^ Tregs and GzmB^+^ CD8^+^ T cells in CCR8^+^ Treg-enriched regions was analyzed using the ROI protocol. Based on the heatmap of CCR8^+^ Tregs from 80 cases, the top five ROIs with the highest cell density were selected, and their average was calculated. Among them, 40 cases with the highest CCR8^+^ Treg accumulation were analyzed. **a.** Correlation plots and regression lines between CCR8^+^ Treg density and total CD8^+^ T cell density are shown. **b.** Correlation plots and regression lines between CCR8^+^ Treg density and GzmB expression proportion in CD8^+^ T cells are shown.

As shown in Figure S5, the spatial analysis-generated dot plot of CCR8⁺ Tregs infiltrating into tumor tissues revealed an uneven distribution within the tumor, with high-density, low-density, and lymphocyte-deficient regions. More CCR8⁺ Tregs were observed to accumulate at the tumor invasion front. To assess the impact of localized CCR8⁺ Treg accumulation on neighboring CD8⁺ T cells within the TME, we used a density heatmap generated by spatial analysis. For each case, we selected five hotspots where CCR8⁺ Tregs were most densely concentrated and analyzed the corresponding GzmB/CD8 stained images from the matched fields. Correlation analysis using hotspot data from the top 40 cases with high CCR8⁺ Treg infiltration showed that [CCR8⁺ Treg (cells/mm²)] was positively correlated with [CD8⁺ T (cells/mm²)], but significantly negatively correlated with [%GzmB⁺ in CD8⁺ T cells] (Figure 4). These findings suggest that in TME regions with high CCR8⁺ Treg accumulation, CD8⁺ T cells also infiltrate these regions although their cytotoxic function may be suppressed.

### Analysis of the distance between CCR8^+^ Tregs and CD8^+^ T cells

**Figure 5:**
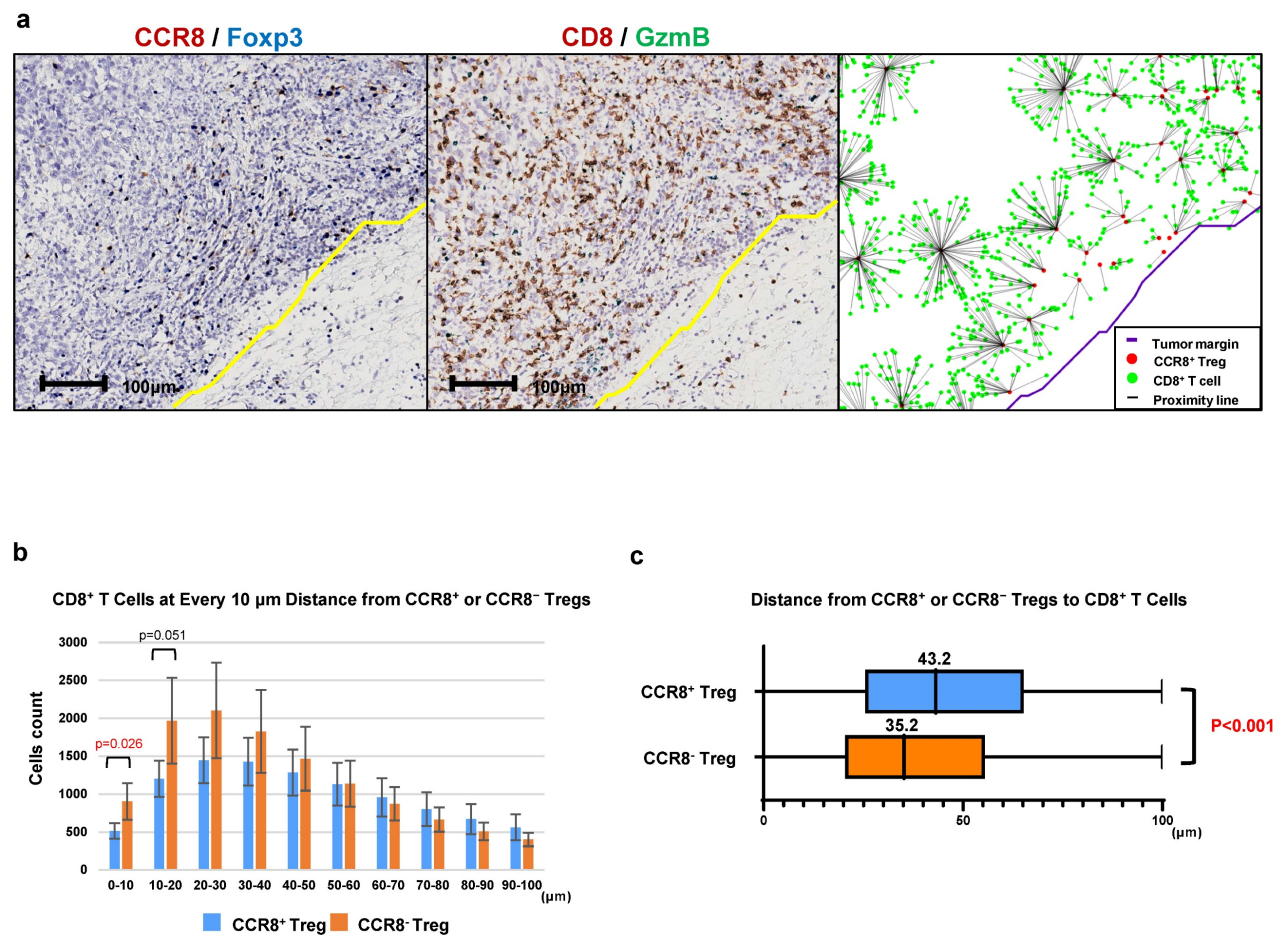
Analysis of intercellular distance between CCR8^+^ Tregs and CD8^+^ T cells. **a.** The number of CD8^+^ T cells closest to CCR8^+^ Tregs was counted at 10-μm intervals up to 100 μm. **b.** Each CCR8^+^ Treg or CCR8^-^ Treg was used as a starting point, and the number of CD8^+^ T cells located within 100 μm was counted at 10-μm intervals from the starting point. **c.** The distance of CD8^+^ T cells from the starting point is shown using box plots.

To investigate the characteristics of CCR8⁺ Tregs within the Treg population, we analyzed the distance between CCR8⁺ Tregs and CD8⁺ T cells in comparison to CCR8⁻ Tregs. As shown in Figure 5, we used CCR8⁺ Tregs or CCR8⁻ Tregs as starting points and counted the number of CD8⁺ T cells at 10-µm intervals up to 100 µm (Figure 5a). The median distance from CCR8⁻ Tregs to CD8⁺ T cells was 35.2 µm, whereas the median distance from CCR8⁺ Tregs to CD8⁺ T cells was 43.2 µm, showing a significant increase in distance between CCR8⁺ Tregs and CD8⁺ T cells (*P* < 0.001) (Figure 5b, c). This finding suggests that CCR8⁺ Tregs suppress the proliferation of CD8⁺ T cells.

## Discussion

In this study, we analyzed the infiltration of CCR8⁺ Tregs and GzmB⁺ CD8⁺ T cells in gastric cancer tissues using IHC dual staining and evaluated their association with tumor stage, prognosis, and spatial distribution. CCR8⁺ Tregs showed a positive correlation with total Tregs and CD8⁺ T cells, indicating that tumors with high Treg infiltration also have a higher number of CD8⁺ T cells. In the prognostic analysis, patients with a low CD8⁺ T cell/CCR8⁺ Treg ratio had a significantly shorter RFS. However, a two-group comparison based on the CD8⁺ T cell/total Treg ratio showed no significant difference in RFS. Patients with high [%CCR8⁺ in Tregs] exhibited a decrease in RFS. These results suggest that the balance between CCR8⁺ Tregs and CD8⁺ T cells is crucial for prognosis; therefore, we analyzed CD8⁺ T cells in the vicinity of CCR8⁺ Tregs to investigate the underlying mechanism. Spatial analysis revealed that CCR8⁺ Tregs were distributed heterogeneously within the tumor and accumulated at the tumor invasion boundary. In regions with high CCR8⁺ Treg density, there was a weak positive correlation between CCR8⁺ Tregs and CD8⁺ T cells, but a significant negative correlation with [%GzmB⁺ in CD8⁺ T cells]. Furthermore, distance analysis between Tregs and CD8⁺ T cells indicated fewer CD8⁺ T cells near CCR8⁺ Tregs than near CCR8^-^ Tregs, suggesting that CCR8⁺ Tregs may control the proliferation of closely infiltrating CD8⁺ T cells. These findings suggest that CCR8⁺ Tregs infiltrate the advanced regions of gastric cancer tumors and suppress antitumor immunity by affecting the surrounding CD8⁺ T cells.

Bulk RNA sequencing analysis of CD3⁺CD4⁺ T cells in renal cancer patients has shown that CCR8 is selectively expressed in tumor-infiltrating activated Tregs 14. Flow cytometry analysis further confirmed that CCR8⁺ Tregs highly express FOXP3 and CD25, along with immunosuppressive molecules such as CTLA4 and CD39, supporting their role as strongly suppressive tumor-localized Tregs. Abundant Treg infiltration in tumors has been associated with poor prognosis 24. More recently, the balance between CD8⁺ T cells and Tregs was reported to be a critical factor for prognosis. Immunohistochemical analyses in patients with ovarian cancer, cervical cancer, or gastric cancer indicated that a high CD8⁺ T cell/Treg ratio correlated with better prognosis 25-27. Furthermore, a high level of CCR8⁺ Tregs was associated with poor prognosis in patients with breast, bladder, or lung cancer 13,16,22,28. In this study, immunohistochemical analysis of gastric cancer tissues revealed that a high CD8⁺ T cell/CCR8⁺ Treg ratio was associated with favorable prognosis, consistent with previous reports indicating that tumors with abundant cytotoxic T lymphocytes and fewer suppressive immune cells had better outcomes. Notably, the CD8⁺ T cell/Treg ratio was not associated with prognosis, suggesting that CCR8⁺ Tregs contribute to poor prognosis by suppressing CD8⁺ T cells in gastric cancer.

Several studies have previously reported the spatial analysis of intercellular distances using IHC staining. In a study analyzing the spatial distribution of infiltrating immune cells in mismatch repair-deficient colorectal cancer treated with anti-PD-1 therapy, the number of PD-1⁺ cells located within 10 μm of PD-L1⁺ cells was found to be a useful predictor of progression-free survival 29. Regarding the spatial distribution of tumor-infiltrating Tregs, an integrated analysis of single-cell RNA sequencing and spatial transcriptomics in colorectal cancer revealed that Tregs co-localized with tumor cells at the tumor-normal interface via the tumor cell-derived signaling molecule midkine, contributing to the formation of an immune-tolerant microenvironment 30. Furthermore, Tregs accumulating at the tumor boundary had higher suppressive activity, with fewer CD4⁺ and CD8⁺ T cells within 100 μm compared with other Tregs, and their presence was associated with suppressed T cell proliferation 31. These findings suggest that the spatial analysis of intercellular distances not only provides insights into the function of individual immune cells but also helps elucidate cell-cell interactions within the TME. Our research showed that CCR8⁺ Tregs localize to the tumor invasion boundary in gastric cancer and can suppress the proliferation and GzmB expression of closely infiltrating CD8⁺ T cells. This is the first report to show, using spatial analysis, that CCR8⁺ Tregs suppress nearby CD8⁺ T cells in gastric cancer.

Anti-CCR8 antibodies are currently undergoing clinical trials for the treatment of cancer. In non-clinical studies using cancer model mice, anti-CCR8 antibodies were shown to deplete tumor-infiltrating Tregs, increase CD8⁺ T cell infiltration within tumors, and enhance CD8⁺ T cell functions by upregulating GzmB and IFN-γ expressions 14,17. Additionally, anti-CCR8 antibodies induced tumor-specific memory T cells, thereby preventing tumor engraftment upon secondary challenge 14. Our current results from the immunohistological analysis of human gastric cancer tissues suggest that CCR8⁺ Tregs may inhibit the proliferation and GzmB expression of surrounding CD8⁺ T cells, leading to the suppression of antitumor immunity. Therefore, anti-CCR8 antibodies might enhance antitumor immunity in gastric cancer tumors where CCR8⁺ Tregs actively suppress immune responses. Future research on anti-CCR8 antibody therapy targeting CCR8⁺ Tregs in gastric cancer is highly anticipated.

This study had several limitations. First, it was an observational study based on a limited number of cases from a single institution. Future prospective validation with a larger sample size is necessary. Second, as mentioned above, despite the potential relationship between the distribution of tumor-infiltrating lymphocytes and histological structure, our analysis did not distinguish between stromal and intra-tumoral regions. This issue will be addressed in future studies.

## Conclusions

This study analyzed the infiltration and distribution of CCR8⁺ Tregs and CD8⁺ T cells in gastric cancer tissues and evaluated their impact on CD8⁺ T cells and patient prognosis. The results suggested that CCR8⁺ Tregs have a suppressive effect on CD8⁺ T cells. Furthermore, a low CD8⁺ T cell/CCR8⁺ Treg ratio was associated with shortened RFS, indicating that the balance between immunosuppression and antitumor immunity plays a role in patient prognosis. These findings suggest that CCR8⁺ Tregs contribute to the suppression of antitumor immunity in gastric cancer, and the selective regulation of Tregs via anti-CCR8 antibodies might represent a novel therapeutic strategy for gastric cancer.

## Supplementary Material

Supplementary figures.

## Figures and Tables

**Table 1:** Clinicopathological characteristics of gastric cancer patients according to CCR8^+^ Treg, CD8^+^ T, and the CD8^+^ T/CCR8^+^ Treg.

Variables	CCR8^+^ Treg high (n = 40)	CCR8^+^ Treg low (n = 40)	*P*-value	CD8^+^ T high (n = 40)	CD8^+^ T low (n = 40)	*P-*value	CD8^+^ T/CCR8^+^ Treg high (n = 40)	CD8^+^ T/CCR8^+^ Treg low (n = 40)	*P*-value
Age in years, median (range)	71 (60—77)	69 (59—74)	0.35	68 (58—74)	71 (62—75)	0.44	68 (58-74)	72 (61-76)	0.24
Sex			0.11			0.82			0.11
Female	12 (30.0)	19 (47.5)		16 (40.0)	15 (37.5)		19 (47.5)	12 (30.0)	
Male	28 (70.0)	21 (52.5)		24 (60.0)	25 (62.5)		21 (52.5)	28 (70.0)	
Tumor location			0.57			0.086			0.57
Upper	13 (32.5)	9 (22.5)		7 (17.5)	15 (37.5)		9 (22.5)	13 (32.5)	
Middle	11 (27.5)	14 (35.0)		16 (40.0)	9 (22.5)		14 (35.0)	11 (27.5)	
Lower	16 (40.0)	17 (42.5)		17 (42.5)	16 (40.0)		17 (42.5)	16 (40.0)	
Histological type			0.82			0.37			**0.0066**
Differenciated type	26 (65.0)	20 (50.0)		21 (52.5)	25 (62.5)		17 (47.5)	29 (72.5)	
Undifferenciated type	14 (35.0)	20 (50.0)		19 (47.5)	15 (37.5)		23 (52.5)	11 (27.5)	
pT			0.67			0.35			0.54
T1	20 (50.0)	24 (60.0)		25 (62.5)	19 (47.5)		23 (57.5)	21 (52.5)	
T2	5 (12.5)	4 (10.0)		5 (12.5)	4 (10.0)		6 (15.0)	3 (7.5)	
T3	10 (25)	6 (15.0)		5 (12.5)	11 (27.5)		6 (15.0)	10 (25.0)	
T4	5 (12.5)	6 (15.0)		5 (12.5)	6 (15.0)		5 (12.5)	6 (15.0)	
pN			0.46			0.77			0.27
N0	25 (62.5)	30 (75.0)		28 (70.0)	27 (67.5)		31 (77.5)	24 (60.0)	
N1	6 (15.0)	2 (5.0)		5 (12.5)	3 (7.5)		4 (10.0)	4 (10.0)	
N2	5 (12.6)	4 (10.0)		4 (10.0)	5 (12.5)		3 (7.5)	6 (15.0)	
N3	4 (10.0)	4 (10.0)		3 (7.5)	5 (12.5)		2 (5.0)	6 (15.0)	
pStage			0.79			0.33			0.11
Stage I	22 (55.0)	25 (62.5)		26 (65.0)	21 (52.5)		25 (62.5)	22 (55.0)	
Stage II	10 (25.0)	8 (20.0)		9 (22.5)	9 (22.5)		11 (27.5)	7 (17.5)	
Stage III	8 (20.0)	7 (17.5)		5 (12.5)	10 (25.0)		4 (10.0)	11 (27.5)	
Lymphatic invasion			0.070			0.65			0.17
Present	27 (67.5)	19 (47.5)		22 (55.0)	24 (60.0)		20 (50.0)	26 (65.0)	
Absent	13 (32.5)	21 (52.5)		18 (45.0)	16 (40.0)		20 (50.0)	14 (35.0)	
Vascular invasion			0.16			**0.019**			**0.019**
Present	23 (57.5)	29 (72.5)		9 (22.5)	19 (47.5)		9 (22.5)	19 (47.5)	
Absent	17 (42.5)	11 (27.5		31 (77.5)	21 (52.5)		31 (77.5)	21 (52.5)	
Preoperative chemotherapy			0.26			0.26			0.26
Treated	10 (25.0)	6 (15.0)		6 (15.0)	10 (25.0)		6 (15.0)	10 (25.0)	
Not treated	30 (75.0)	34 (85.0)		34 (85.0)	30 (75.0)		34 (85.0)	30 (75.0)	

Data are presented as n (%) unless noted otherwise. Abbreviations: CCR8, CC motif chemokine receptor 8; Treg, regulatory T cell Bolded *P*-values indicate statistical significance (*P* < 0.05)

## Abbreviations

CCR8: CC motif chemokine receptor 8
FoxP3: Forkhead box protein 3
GzmB: Granzyme B
ICI: immune checkpoint inhibitors
IHC: immunohistochemistry
PD-L1: Programmed death ligand 1
PD-1: Programmed cell death 1
RFS: Recurrence-free survival
ROI: Regions of Interest
Treg: Regulatory T cell
WTA: Whole tumor area
