# Supplementary material for: The immunosuppressive role of CCR8+ Tregs in gastric cancer
Source: J Cancer. 2025 Nov 3;16(15):4390–9. doi: 10.7150/jca.121363 (PMC12664733; doi:10.7150/jca.121363)
Supplement: Supplementary file 1 — Supplementary figures. [file jcav16p4390s1.pdf]

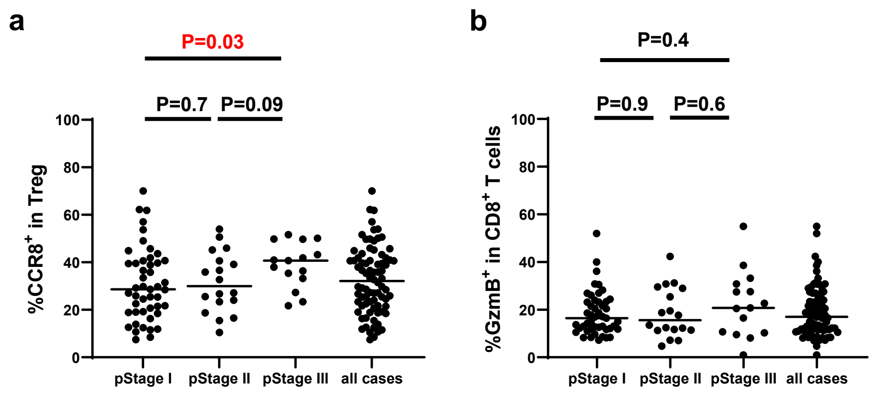

Supplementary Fig. 1 Proportions of CCR8 in Tregs, GzmB in $CD8^+$ T cells, and $CCR8^+$ Tregs among $CCR8^+$ cells. a. Proportion of CCR8 expression in Tregs by pathological stage is shown. b. Proportion of GzmB in $CD8^+$ T cells by pathological stage is shown

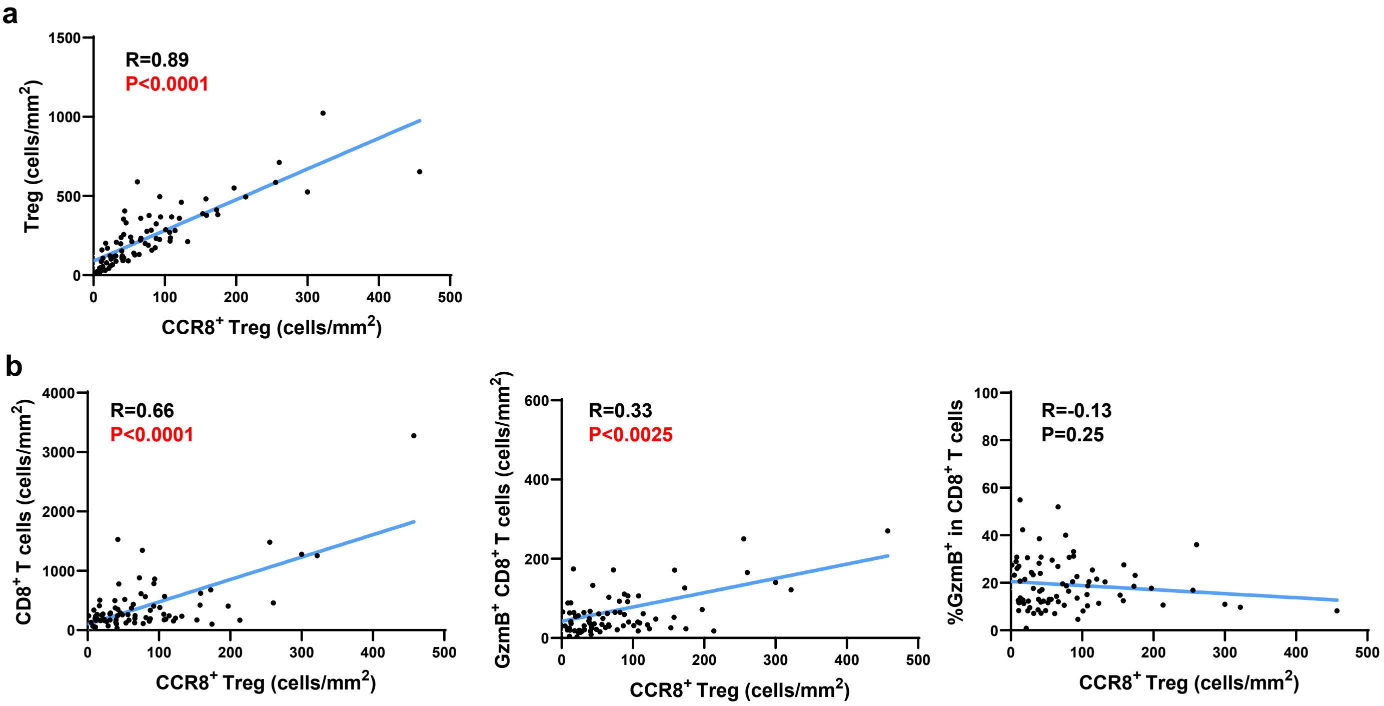

Supplementary Fig. 2 Association of CCR8⁺ Tregs with GzmB⁺ CD8⁺ T cell parameters. a. Correlation plots and regression lines between CCR8⁺ Treg density and total Treg density are shown. b. Correlation plots and regression lines between CCR8⁺ Treg density, total CD8⁺ T cell density, GzmB⁺ CD8⁺ T cell density, and GzmB expression proportion in CD8⁺ T cells are shown

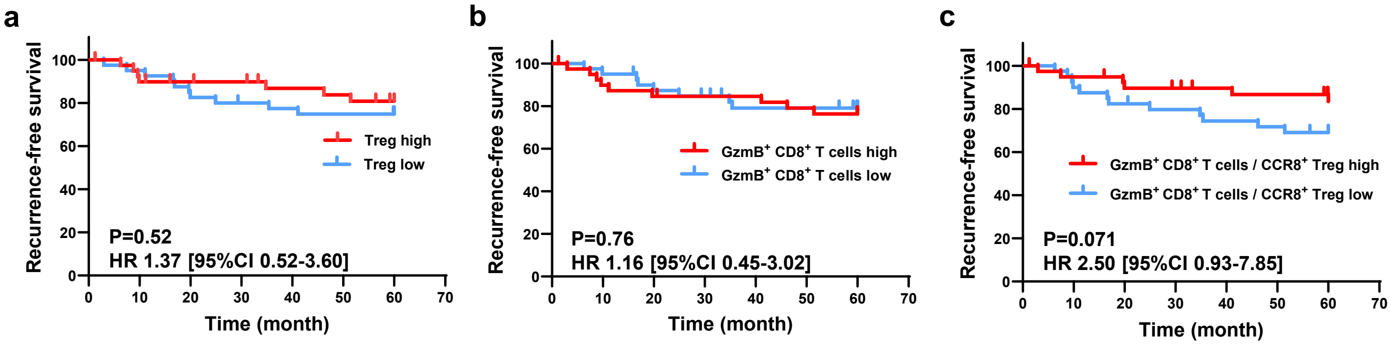

Supplementary Fig. 3 Evaluation of the prognostic impact of $CCR8^+$ Treg and $GzmB^+$ $CD8^+$ T cell infiltration. a. Patients were divided into high-density and low-density groups based on the median total Treg density, and the 5-year survival rates were compared between the two groups. Similarly, the comparison was also performed based on (b) the density of $GzmB^+$ $CD8^+$ T cells and (c) the ratio of $GzmB^+$ $CD8^+$ T cell density to $CCR8^+$ Treg density

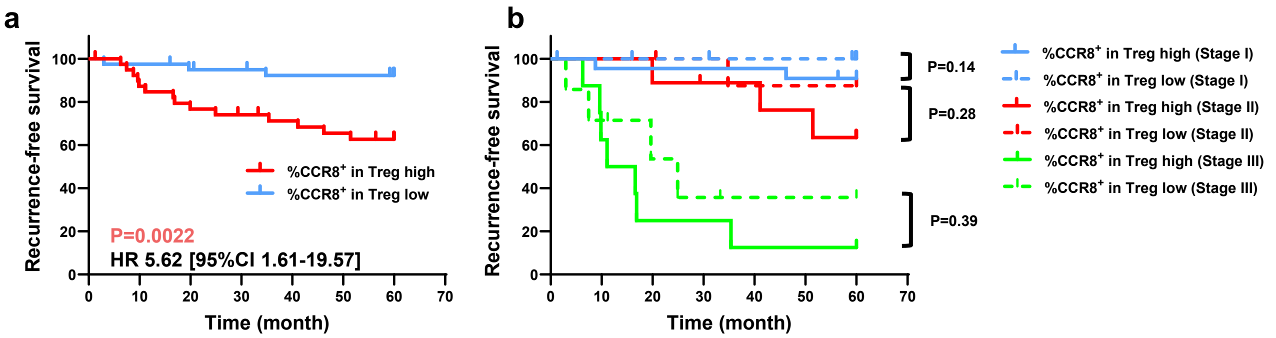

Supplementary Fig. 4 Evaluation of the prognostic impact of proportions of CCR8 expression in Tregs. a. Patients were divided into high and low CCR8 expression groups based on the median CCR8 expression rate in Tregs, and the 5-year survival rates were compared between the two groups. b. Similar analysis was performed for each pStage

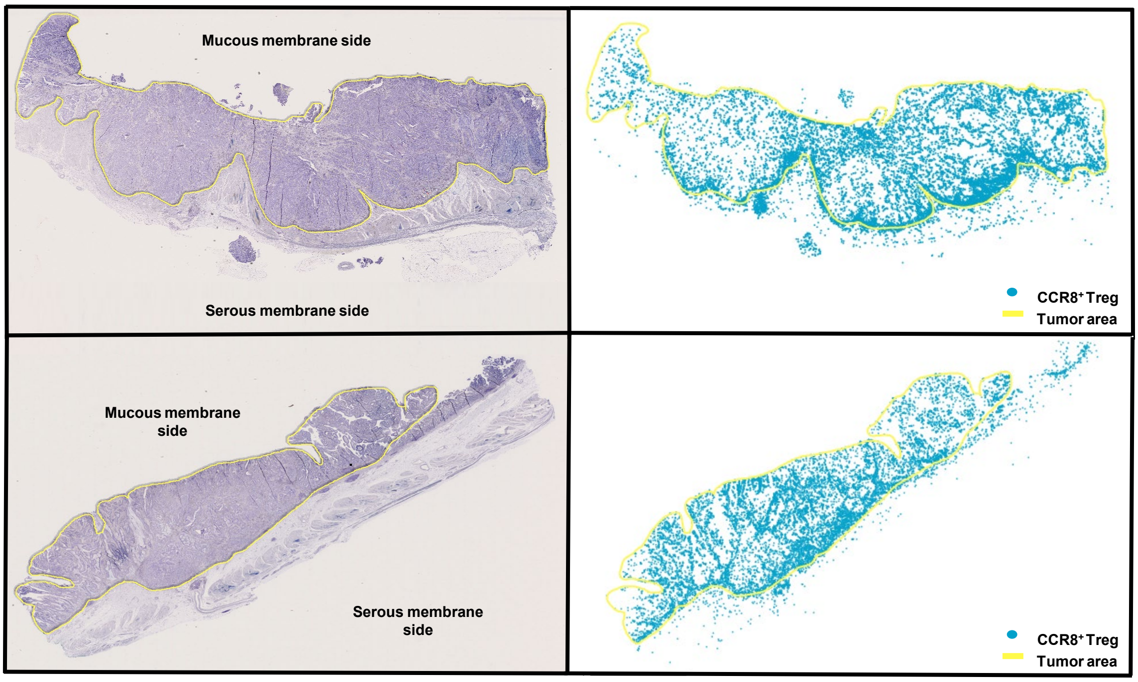

Supplementary Fig. 5 Representative dot plots of $CCR8^+$ Tregs from two cases analyzed by immunohistochemical image analysis. The image on the left shows a double immunostained for CCR8/Foxp3, with the tumor area outlined by a yellow line. The image on the right shows a dot plot of $CCR8^+$ Tregs in the tissue depicted on the left, where the yellow line outlines the tumor area
